# Real‐time quantification and supplementation of bioreactor amino acids to prolong culture time and maintain antibody product quality

**DOI:** 10.1002/btpr.2894

**Published:** 2019-08-28

**Authors:** David N. Powers, Yifan Wang, Erica J. Fratz‐Berilla, Sai Rashmika Velugula‐Yellela, Brittany Chavez, Phillip Angart, Nicholas Trunfio, Seongkyu Yoon, Cyrus Agarabi

**Affiliations:** ^1^ U.S. Food and Drug Administration, Center for Drug Evaluation and Research, Office of Product Quality, Office of Biotechnology Products, Division of Biotechnology Review and Research II Silver Spring Maryland; ^2^ U.S. Food and Drug Administration, Center for Drug Evaluation and Research, Office of Testing and Research, Division of Product Quality Research Silver Spring Maryland; ^3^ Sartorius Stedim North America Inc Corporate Research Bohemia NY; ^4^ Department of Chemical Engineering University of Massachusetts Lowell Massachusetts

**Keywords:** amino acids, bioprocessing, glycosylation, MVDA, PCA, process analytical technology

## Abstract

Real‐time monitoring of cell cultures in bioreactors can enable expedited responses necessary to correct potential batch failure perturbations which may normally go undiscovered until the completion of the batch and result in failure. Currently, analytical technologies are dedicated to real‐time monitoring of bioreactor parameters such as pH, dissolved oxygen, and temperature, nutrients such as glucose and glutamine, or metabolites such as lactate. Despite the importance of amino acids as the building blocks of therapeutic protein products, other than glutamine their concentrations are not commonly measured. Here, we present a study into amino acid monitoring, supplementation strategies, and how these techniques may impact the cell growth profiles and product quality. We used preliminary bioreactor runs to establish baselines by determining initial amino acid consumption patterns, the results of which were used to select a pool of amino acids which gets depleted in the bioreactor. These amino acids were combined into blends which were supplemented into bioreactors during a subsequent run, the concentrations of which were monitored using a mass spectrometry based at‐line method we developed to quickly assess amino acid concentrations from crude bioreactor media. We found that these blends could prolong culture life, reversing a viable cell density decrease that was leading to batch death. Additionally, we assessed how these strategies might impact protein product quality, such as the glycan profile. The amino acid consumption data were aligned with the final glycan profiles in principal component analysis to identify which amino acids are most closely associated with glycan outcomes.

## 1 INTRODUCTION

Nutrient‐rich bioreactor media is crucial for optimal production from cultured Chinese hamster ovary (CHO) cells that have become the preferred platform for the manufacturing of therapeutic proteins such as monoclonal antibodies (mAb).^1^, ^2^ As nutrients such as amino acids are consumed during the manufacturing process and metabolites are produced, analytical methods to determine how their changing concentrations impact critical quality attributes (CQA) such as product glycosylation are essential.^3^ For this purpose, we developed a process analytical technology (PAT) enabling application that allows for rapid assessment of bioreactor amino acid levels. For the sake of speed and simplifying sample preparation, this chromatographic approach measures nonlabeled underivatized amino acids using a mass spectrometer. This allows us to be able to quantitate amino acid concentrations in the bioreactor media in near real time and support customized control of individual nutrient species. After the bioreactor run was completed, we performed glycan characterization and measured additional product quality characteristics such as charge variants and size variants, permitting us to associate specific amino acids with their estimated effect on the final antibody quality profile. Our results demonstrate the feasibility of PAT tools in a manufacturing setting to aid bioprocessing and support the development of advanced feeding strategies to control product quality.

Our research addressed the need to better understand how complex input variables within the biomanufacturing process affect product quality. CQAs are physical, chemical, biological, or microbiological properties that must be within specified ranges to ensure the desired product quality, and in the case of mAb products one particularly important CQA is the distribution of product glycoforms.^4^, ^5^, ^6^, ^7^ *N*‐linked glycosylation may affect various therapeutic properties of the antibody, from effector function, immunogenicity, stability, and clearance rate.^8^ IgG antibodies feature conserved *N*‐glycosylation sites at Asn^297^ in the constant region of the Fc heavy chain, which influence effector binding to downstream molecules such as Fcγ receptors that mediate antibody‐dependent cellular cytotoxicity (ADCC), a critical mechanism for many biotherapeutics.^9^ Even for antibodies where the mechanism of action is not through ADCC, an abnormal glycan profile can affect efficacy through alteration of the immunogenicity, stability, or clearance rate.

The mechanisms by which glycoforms can affect antibody product performance have been experimentally characterized, such as how fucose sterically inhibits interaction with the Fcγ receptor and reduces ADCC‐mediated activity.^10^ Conversely, how the cell growth and media conditions within the bioreactor impact the glycan profile are not well understood. Bioreactor culturing conditions have been established in their ability to change product quality outcomes: specifically temperature, pH, and agitation rate have been shown to have small effects on the final glycan profile.^11^ However, the role of overlooked nutrients in the media such as sugars, amino acids, and metals have been only minimally characterized with respect to how they can affect protein production and efficacy.^12^

Amino acids are crucial for antibody production since they are the building blocks of the primary protein sequence.^13^ As such, supplying the cells with sufficient levels of amino acids is needed to avoid protein anomalies, such as amino acid misincorporation which changes the primary sequence of the protein.^14^ Khetan et al discovered that when asparagine was depleted from the bioreactor media, serine was misincorporated in asparagine's place at rates as high as 3%. Amino acids also serve as potential energy sources for the cell, such as glutamate via glutaminolysis. Thus, it is possible that amino acid depletion could affect more than the primary sequence of the protein, such as the posttranslational modifications and the chemical structure of the molecule. Regarding these considerations, we developed an at‐line amino acid quantification method that may be a useful tool to monitor upstream bioprocessing. Specifically, we are studying the possibility that replenishment of selected amino acids could help maintain the product quality of the bioreactor cell culture. Altered supplementation of amino acids in cell culture can modify the critical quality attributes of the antibody, including glycosylation.^15^ These changes are not limited to antibody products: an increase of recombinant human erythropoietin with decreased sialylation has been attributed to the addition of amino acids as feeds during cell culture.^16^ In a study of tissue plasminogen activator produced in ammonium‐stressed CHO cells, the addition of amino acids leads to increased glycosylation site occupancy by mature glycoforms.^17^ However, contemporary studies have not applied these supplementation strategies with real time quantification which would help to better target and understand specific relationships between the nutrient addition and product quality.

Current amino acid analysis is divided into two approaches: derivatized and underivatized.^18^ Derivatized methods require chemical modification of the amino acids so they can be detected via spectroscopy, typically with an ultraviolet (UV) or fluorescence detector. Underivatized amino acid analysis does not require a chemical reaction, lending itself to be more easily implemented in PAT development due to simpler sample preparation. Underivatized analysis requires mass spectrometry since native amino acids cannot currently be differentiated directly with spectrometric methods. We present here an underivatized at‐line method using mass spectrometry for quantification of amino acids that allowed us to assess depleted amino acids in near real time, using this information to determine which amino acids are consumed most quickly in the bioreactor and the relative importance of each amino acid toward the resulting glycan profile.

## 2 MATERIALS AND METHODS

### 2.1 Cell culture reagents

An in‐house CHO DG44 cell line cultured in CD OptiCHO (Thermo Fisher Scientific, Waltham, MA) media was used for this experiment. The growth and metabolic activity of the cell line have been well documented based on previous bioreactor runs (data not shown). The media was initially supplemented with 8 mM l‐glutamine (Corning, Manassas, VA) and 1X Penicillin/Streptomycin (Corning, Oneonta, NY). Glucose is contained in the chemically defined media at 5–6 g/L. Cys, Asn, Met, His, Trp, Thr, Tyr, and Pro were used in varying combinations as amino acid supplementations. The amino acids were dissolved in 0.3 N HCl (Thermo Fisher Scientific, Waltham, MA) before being added to the bioreactors. During the process, when glucose was less than 1 g/L and glutamine was less than 1 mM, glucose and l‐glutamine were supplemented as a bolus to bring back the levels to 5 g/L and 8 mM, respectively.

### 2.2 Seed train

This experiment used a previously described recombinant CHO DG44 cell line that expresses a model chimeric IgG1.^19^ For all the reactors subjected to feed strategies 1 through 3, 5, and 6, the seed train was started by thawing 1 vial (1 ml) of banked cells (7 × 10^7^ viable cells/ml) into 200 ml of 37°C CD OptiCHO media (Life Technologies, A11222) supplemented with 1X Penicillin/Streptomycin (Corning, 30‐001‐Cl) and 8 mM l‐glutamine (Corning, 25‐005‐CV) in a 1 L spinner flask (Corning, 4500‐1L). Incubator CO_2_ was set at 5%, temperature was set at 37°C and stir speed was kept at 65 RPM. Scale‐up procedures were used until 4 1 L spinner flask cultures with >2 × 10^6^ cells/ml were created.

Alternatively, for the reactors subjected to feed strategies 4 and 7, the seed train was started by thawing a 1 ml vial of banked cells (7 × 10^7^ viable cells/ml) into 50 ml of 37°C media contained in a 125 ml disposable shake flask (Corning, PBV12‐5). Upstream scale‐up procedures were followed until the cells were inoculated into a 2 L cell bag (GE Healthcare, CB0002L10‐31) on a GE ReadyToProcess WAVE™ 25 rocker system at 1 × 10^6^ cells/ml in 750 ml. Gas mix flow rate was set to 0.3 L/min, pH was set to 7.1 (CO2/base‐controlled), DO was set to 50% air saturation, and rocking speed and angle were 20 rpm and 6°, respectively. After 1 week, the cells reached 5.7 × 10^6^ cells/ml and were prepared for inoculation.

On the day of inoculation, cell culture fluid from either the spinner flasks or WAVE bag was transferred to disposable, sterile 250 ml conical tubes and centrifuged at 800 rpm for 10 min. Supernatant was slowly decanted and cell pellets were resuspended in 5 ml of fresh, prewarmed media and pooled for bioreactor inoculation.

### 2.3 Bioreactor processing conditions

A BIOSTAT B‐DCU II (Sartorius Stedim Biotech, Goettingen, Germany) bioreactor system with two to four 5 L vessels was run in batch or fed‐batch mode for 5–10 days (120–240 hr). Set points for the culture processes (Table 1) were maintained automatically by the controller. Culture foaming was reduced when foam reached foam sensors on the head plate of the reactor and additionally by manual addition as needed using a 3% EX‐CELL gamma‐irradiated antifoam emulsion (Sigma‐Aldrich, 59920C‐1B). Each reactor was equipped with a FUTURA 12 mm biomass probe with FUTURA head amplifier (ABER Instruments, 2330‐00) to obtain viable cell density measurements in real‐time. Bolus feeds of glucose, glutamine, and amino acid blends were supplemented as shown in Table 2.

**Table 1:** Summary of culture process set points and controls

Culture parameter	Setpoint or description
Culture volume	4 L
Sparge rate	0–30 ccm
Dissolved oxygen	50%
Dissolved oxygen control	Air, O_2_, and CO_2_
Agitation rate	150 rpm
Temperature	37°C
Inoculation density	3 × 10^5^–1 × 10^6^ cells/ml
pH	7.1
pH control	0.5 M NaOH and CO_2_

**Table 2:** Feeding and amino acid supplementation strategies for nine bioreactors

Feed strategy	Number of bioreactors	Glucose feed initiation (g/L)	Glucose feed added (g/L)	Glutamine feed initiation (mM)	Glutamine feed added (mM)	Amino acid blends (first, second)^a^	Amino acid addition time (hour)
1	1	–	–	–	–	–	–
2	2	<1.5	1	1	2	–	–
3	2	<0.2	6	<1	8	–	–
4	1	<2.5	2.5	4	4	A, A + B	127, 221
5	1	<1	7	1	9	B, C	105, 166
6	1	<1	7	1	9	A, A + B	119, 170
7	1	<2.5	2.5	4	4	B, D	77, 170

a Amino acid blend A: Tyr, Cys, Pro, Asn; amino acid blend B: Met, His, Trp, Thr; amino acid blend C: Cys, Asn; amino acid blend D: Tyr, Pro.

### 2.4 Cell counts and nutrient analysis

Samples were run on the BioProfile FLEX analyzer (Nova Biomedical) either automatically using the BioProfile FLEX on‐line autosampler or taken manually to measure viable cell density (VCD), pH, glutamine, glucose, lactate, glutamate, and ammonium. Additionally, cell count was monitored using a biomass probe as mentioned previously. Samples were taken anywhere from every 4 hr to once daily, depending on the run. Additional sample volume obtained for amino acid analysis was clarified by centrifugation at 300*g* for 5 min at 4°C and sterile filtered using 0.22 μm PVDF filters. Cell‐free samples were frozen and stored at −20°C until future analysis.

### 2.5 Downstream mAb purification

The methods used to purify and concentrate the antibody produced by the bioreactors were described previously.^20^

### 2.6 Amino acid characterization by LC–MS

For amino acid analysis by LC–MS, crude bioreactor media was centrifuged and passed through a 0.22 μm filter. A perchloric acid cleanup was used to remove protein and particulate matter, which involved mixing filtered bioreactor media with 0.4 N HClO_4_ at a 1:1 ratio and centrifuging at 1,962*g* for 5 min at RT.^20^ The clarified media was collected to be analyzed by LC–MS.

A Waters Xevo G2 Q‐ToF (run in ESI positive sensitivity mode) coupled to a Waters ACQUITY UPLC I‐Class was used for analysis. We used an Intrada Amino Acid column (Imtakt USA) (100 × 2 mm, 3 μm particles) to perform normal phase chromatography and separate the amino acids. The buffers used were A: acetonitrile + 0.1% formic acid and B: 100 mM ammonium formate, with a flow rate of 0.6 ml/min, a gradient time of 15 min, and column temperature of 40°C. Amino Acid Standards (Agilent) were utilized to generate a calibration curve (9 to 900 pmol/μl) in the QuanLynx software (Waters), which was used to calculate the concentrations of amino acids detected in the prepared bioreactor media samples. Media samples were run in triplicate, with error bars indicating *SD*s. Injection order was randomized to eliminate order bias. Additional information on this method can be found in past work.^20^

### 2.7 Glycan characterization

The GlycoWorks method used to label and quantitate glycans isolated from the harvested antibodies was described previously.^20^ The glycan profiles were only evaluated for the final harvest product for each bioreactor that was run.

### 2.8 Charge variant analysis

The relative mAb charge variant distribution was measured with the Perkin Elmer GXII Touch HT, utilizing the DNA 5K/RNA/CZE Chip (Cat# 760435) and Protein Charge Variant Kit reagents (Cat# CLS760670). In brief, purified mAb was desalted with a Zeba desalting column (Thermo Fisher Scientific Cat# 89883) and 50 μg (25 μl at 2 mg/ml) of protein was labeled and analyzed in triplicate. The chip was prepared with reagents at pH 7.2 and the assay time was 90 s. Relative charge distributions were determined with GXII reviewer software. All basic and acidic species were grouped together for analysis.

### 2.9 Size variant analysis

Samples were analyzed using the Perkin Elmer Labchip GXII Touch HT with a Labchip HT Protein Express Chip (Cat# 760499) and Protein QC Reagent Kit reagents (Cat# CLS960014). The specified analysis program was the HT Protein QC 250. Samples were analyzed in triplicate at 2.5 μg (2.5 μl at 1 mg/ml) and denatured at 70°C for 10 min with 7.7 mM Iodoacetamide (ThermoFisher Scientific Cat# 90034), nonreducing conditions, or with 30.7 mM DTT (Sigma‐Aldrich Cat# 10197777001), reducing conditions. Relative peak areas were determined using the GXII reviewer software.^20^

### 2.10 Multivariate data analysis

The following amino acids were included in the principal component analysis: Phe, Trp, Leu/Ile, Met, Pro, Tyr, Val, Ala, Thr, Glu, Asp, Gly, Ser, Gln, Asn, His, Lys, Arg, and HyPro. Cys was not included due to the difficulty in combining data between the Cys and Cys_2_ (cystine) forms. To balance between the measurement resolution of the amino acids’ concentration and auto‐correlation associated with the time‐dependent amino acid profiles, we selected nine sampling points that are evenly distributed throughout the experiment to include in the PCA model. Therefore, the PCA model included 1,539 data points from the nine experimental batches.

The data for the principal component analysis was analyzed using Unscrambler X 10.4 (Camo, Oslo, Norway). Since the variation of each amino acid has different scale, the data was first pretreated by scaling with *SD* so that data after pretreatment has a *SD* of 1. The employed pretreatment is a commonly utilized processing for most machine learning estimators when comparing similarities between samples based on certain distance measures.^21^

In principal component analysis, the model represents the pretreated amino acids profile (X) in a reduced dimension (principal component space) such that the major axes of variability are identified. The dataset X can then be decomposed, based on the equation below, into a set of scores (T) and loadings (P), while the remaining variability is modeled as random error (*ε*).

$$X=TP^{T}+\varepsilon$$

The columns *T* represent principal component (PC) scores of an amino acid at certain time point in the projected space; loadings *P* represent the significance of an amino acid in each principal component. Both *T* and *P* are obtained from eigenvectors and eigenvalues of the covariance matrix of *X*. The eigenvectors are orthogonal to each other, and each is associated with the value that explains the proportion of variability in the *X*.

In this study, the calculation of principal components was based on nonlinear iterative partial least squares (NIPALS) algorithm because of its robustness of handling missing data. Cross‐validation was used for model evaluation and as a tool to help in the model development. Cross‐validation groups were established based on the nine experiment batches to perform a leave‐one‐class‐out challenge to the model.

## 3 RESULTS AND DISCUSSION

**Figure 1:**
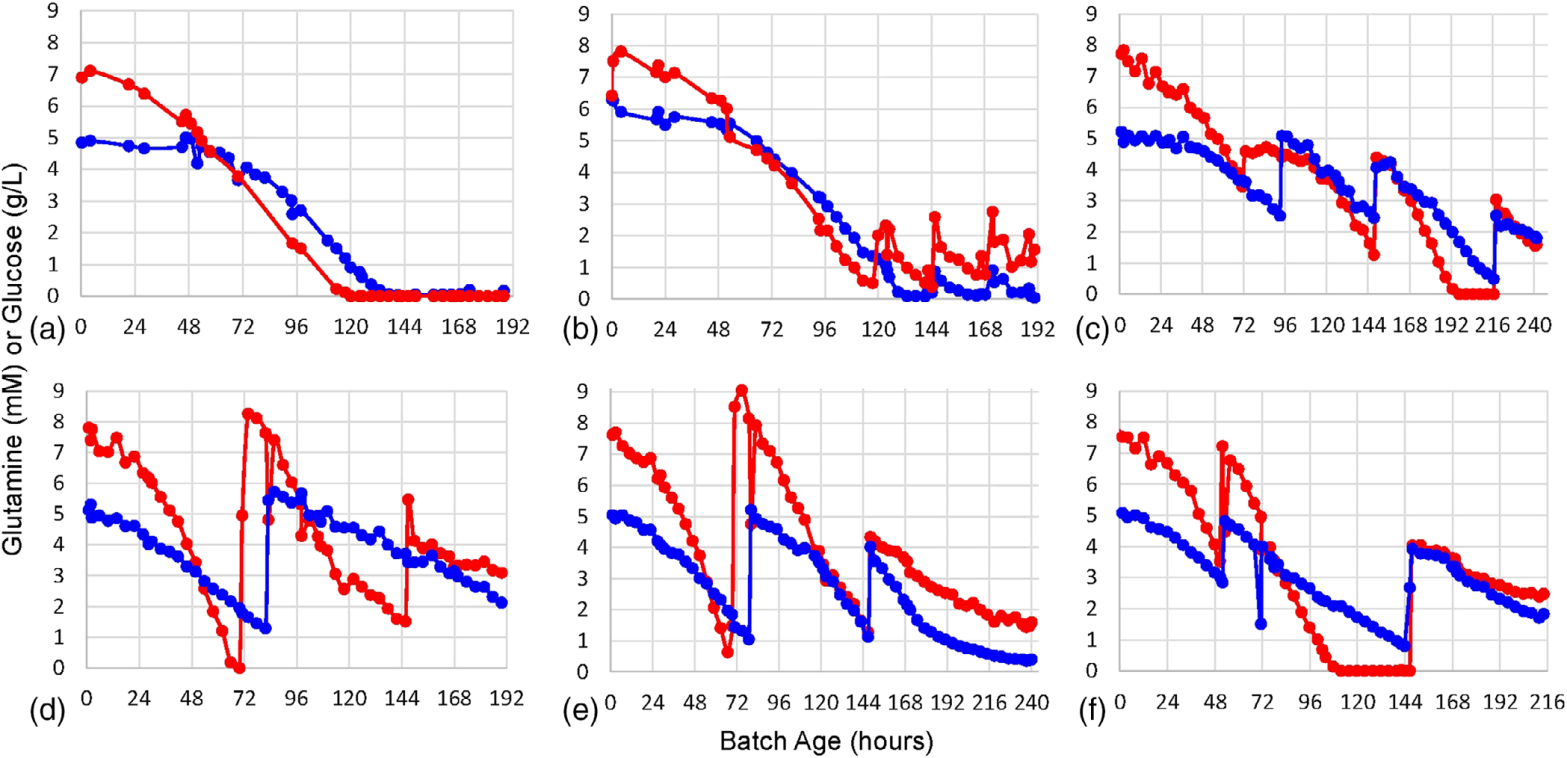
Glutamine and glucose feed strategies. Glutamine measurements (mM) are shown in red and glucose measurements (g/L) are shown in blue. The graphs are organized by feed strategy: (a) batch strategy 1, (b) fed‐batch strategy 2, (c) fed‐batch and amino acid supplementation strategy 4, (d) fed‐batch and amino acid supplementation strategy 5, (e) fed‐batch and amino acid supplementation strategy 6, (f) fed‐batch and amino acid supplementation strategy 7

**Figure 2:**
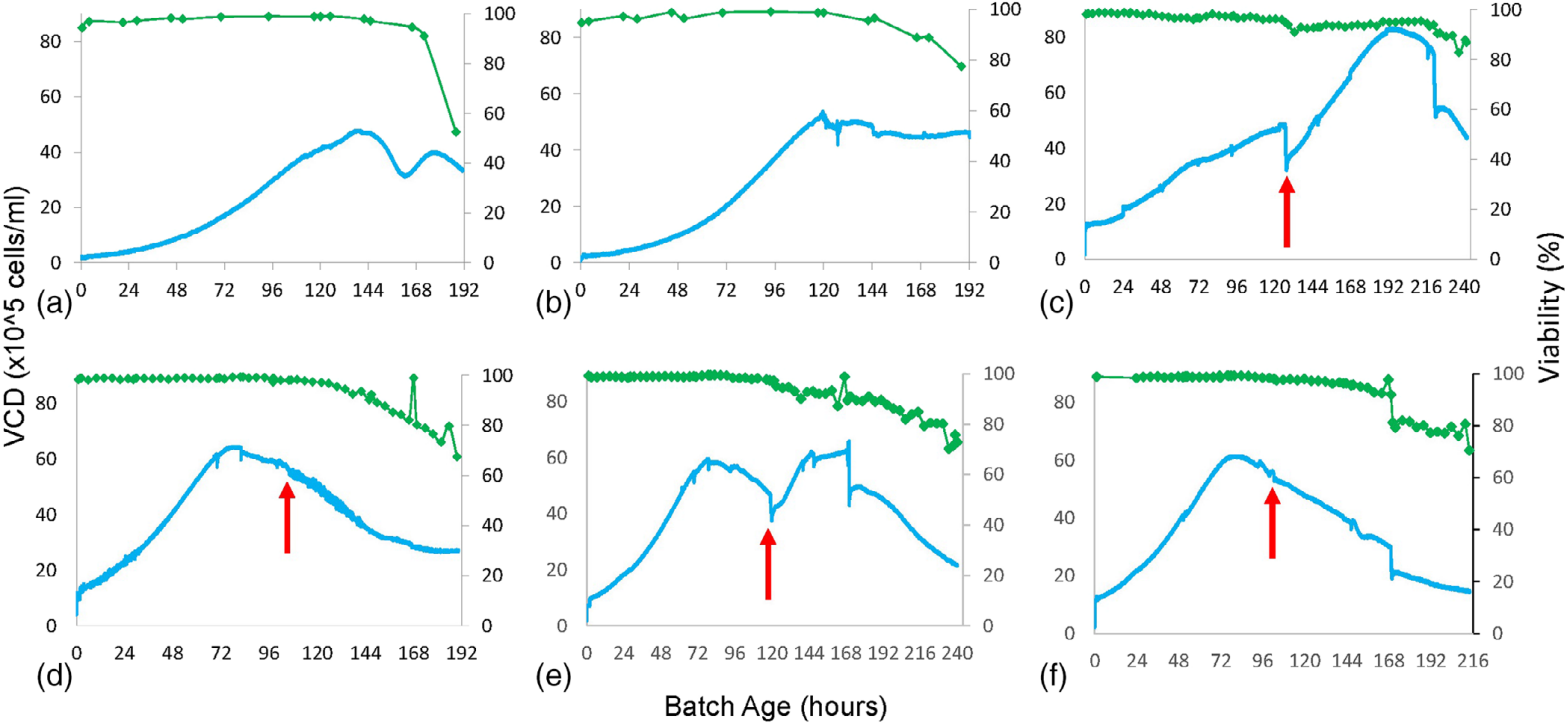
Growth profiles for different feeding strategies. VCD (10^5^ cells/ml) is shown in blue as measured by biocapacitance and green data points are viability (%) measurements. The red arrows indicate when the first amino acid supplementation event occurred. (a) batch strategy 1, (b) fed‐batch strategy 2, (c) fed‐batch and amino acid supplementation strategy 4, (d) fed‐batch and amino acid supplementation strategy 5, (e) fed‐batch and amino acid supplementation strategy 6, (f) fed‐batch and amino acid supplementation strategy 7

Initially, we ran multiple bioreactors to serve as test runs and establish baseline conditions prior to experimentation with amino acid supplementation strategies (Table 2). One bioreactor was run in batch mode (Feed strategy 1), while four others were run in fed‐batch mode using two different sets of glucose and glutamine levels (Feed strategies 2 and 3). Glutamine is an amino acid that is already actively maintained and monitored in standard bioreactor setups, as it is metabolized for energy under high growth conditions where large amounts of proteins are produced. Details on the culture process set points and controls for the runs can be found in the Materials and Methods. Representative glucose and glutamine measurements for these preliminary runs are depicted in Figure 1a,b, and growth profiles in Figure 2a,b.

These bioreactor runs proceeded as expected with glucose and glutamine steadily consumed in the batch mode vessel, while these nutrients were added to the fed‐batch vessels when the nutrient concentrations decreased below predetermined thresholds. The fed‐batch conditions expectedly prevented significant losses in viable cell density (VCD) or viability, but the batch conditions in Figure 2a resulted in large losses of these cells after 168 hr of culture time. We quantified the nutrient depletion in these cultures and surmised that this is likely a cause for the loss in VCD.

We quantified amino acid levels in these preliminary bioreactor runs to better understand the expected consumption patterns and to formulate amino acid blends to use in the final four bioreactors. To support the addition of amino acid blends during the runs, near‐real time analytics were established to allow for rapid quantification of amino acids in the bioreactor vessels. This analytical method for crude bioreactor media was developed with future on‐line real‐time PAT implementation in mind, which necessitates minimal sample preparation that is fast and avoids derivatization when possible. With our normal phase liquid chromatography mass spectrometry (LC–MS) based method, crude media can be collected from the bioreactor, processed in less than 10 min and run on a 15‐min gradient for complete amino acid characterization in near‐real‐time. Only 10 μl is required per replicate, allowing this method to be used for cultures with small working volumes as well, such as microbioreactors. We used the near‐real‐time measurements while adding amino acid blends to the final four bioreactors to validate the impacts of amino acid spikes on amino acid concentrations, while the less critical time points that were in‐between spiking events were collected and analyzed later.

**Figure 3:**
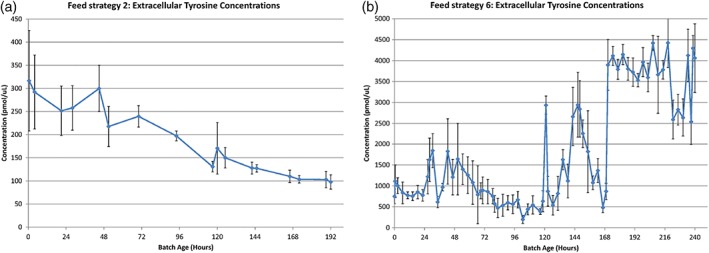
Representative amino acid consumption profile (tyrosine). The consumption and supplementation of tyrosine (pmol/μl) in two representative culture lifetimes (hours). (a) Tyrosine consumption in fed‐batch feed strategy 2, (b) tyrosine consumption and supplementation in feed strategy 6

A representative example of the nonderivatized amino acid concentrations measured by mass spectroscopy is displayed in Figure 3. As shown in Figure 3a, the culture concentrations of tyrosine (Tyr) decreased gradually over the full course of the fed‐batch mode run. Due to this depletion, tyrosine was picked as an amino acid for supplementation in the final batches. The other amino acids used in the supplementation blends were selected with this reasoning as well: cysteine (Cys), proline (Pro), asparagine (Asn), methionine (Met), histidine (His), tryptophan (Trp), and threonine (Thr). To help us differentiate how each amino acid might potentially affect the culture, they were added in different blends at two times during the bioreactor run (Table 2). The total pool of eight amino acids was divided into two halves and used for the first amino acid supplementation event ((A) Tyr, Cys, Pro, and Asn or (B) Met, His, Trp, and Thr) and occurred between 77 and 127 hr after inoculation. Rather than supplementing amino acids at a predetermined timepoint, we decided to supplement when the conditions of the bioreactors were at a similar growth state. The first amino acid addition event for each bioreactor was timed to occur when the VCD value of the bioreactor had been decreasing for approximately 24 hr (except in the case of Figure 2c, where the amino acid blend was added before this occurred due to the slower growth profile of this bioreactor culture) and the remaining concentrations of the amino acids of interest were determined to be depleted by mass spectrometric analysis. The first addition events are indicated by red arrows in Figure 2c–f. The second amino acid addition occurred between 166 and 221 hr after inoculation and was timed to determine its effect on the growth profiles of the cultures. The second amino acid supplementation used one of three possible combinations: (A + B), (C) Cys and Asn or (D) Tyr and Pro.

Figure 2c–f depicts the VCD during the experimental bioreactor run where four 5 L stainless steel bioreactors were operated with periodic quantification of amino acid concentrations. The specifics for the feeding conditions of these vessels, as well as for the amino acid blends, can be found in Table 2.

As shown in Figure 2c–f, the amino acid blends had markedly different effects on the VCD profiles of the bioreactors. By observing how the VCD changed after the first amino acid addition events (indicated by the red arrows), we can identify large differences in culture responses. For example, while the amino acid supplementation in feed strategies 5 and 7 (Figure 2d,f) appear to have had no effects on VCD growth, there was a dramatic effect on VCD found in feed strategies 4 and 6 (Figure 2c,e). Feed strategies 4 and 6 both used amino acid blend A containing Tyr, Cys, Pro, and Asn as the first addition. This resulted in a sharp increase in VCD growth, as demonstrated by the steepness of the VCD line after the amino acid feeding event. Even more surprisingly when amino acid blend A was added in feed strategy 6 (Figure 2e) and the VCD was already decreasing, the amino acid addition was able to reverse this loss. This indicates that the blend containing Tyr, Cys, Pro, and Asn has the beneficial effect of prolonging bioreactor production time and increasing the VCD. This combination of amino acids, however, did not have any effect when it was added as a second supplementation event (amino acid blend A + B) that occurred 50+ hours after the first amino acid supplementation.

Since past studies have shown that changing the growth conditions of the cells can affect product quality, the next step was to characterize the protein product and determine if any significant changes had occurred. To address this, the final antibody product from these cultures was collected, purified, and analyzed to determine the charge variant profile, size variant profile, and glycan profile.

Charge variant and size variant analysis using microfluidic electrophoretic approaches performed on the product antibody revealed small differences between feeding strategies 4 and 6 (which used amino acid blends A and A + B) and feeding strategies 5 and 7 (which used amino acid blends B, C, and D). Amino acid blend A, which was responsible for the boost in VCD in the bioreactors, also produced a small decrease in basic species (Table 3). Another important distinction for the antibody products produced between by the amino acid blends was the formation of a second peak (labeled as Peak 2 in Tables 4 and 5) during purification that was only observed in cultures supplemented by amino acid blend A. This secondary peak was isolated and analyzed alongside the primary peak for size analysis and glycan analysis. Size variant analysis of the intact mAb product revealed that the products were comparable (Peak 1), while the secondary peak was likely containing some level of contamination (Table 4) as size variant analysis is typically used as a measure of purity. Reduced size variant analysis was performed to get a better understanding of how the light and heavy chains constituting the mAb might have been affected by different amino acid blends during upstream processing (Table 5). The main peak showed no differences due to the amino acid blends as the percentages of light and heavy chain were not changed between feeding strategies. However, the secondary peak for feed strategies 4 and 6 affirmed that the species in this peak were different, with a significantly lower amount of heavy chain than in the main peak and a large amount of unknowns. Altogether, the charge and size variant analyses supported that there were small differences in the charge variant profile that likely would not have a significant effect on the therapeutic properties.

**Table 3:** Charge distribution of purified antibody from the amino acid supplemented bioreactor run

Average of % relative amount	Feed strategy 4	Feed strategy 5	Feed strategy 6	Feed strategy 7
Basic species	13.3 ± 0.1	12.0 ± 0.2	13.9 ± 0.1	12.0 ± 0.2
Main peak	56.9 ± 0.4	57.9 ± 0.7	57.7 ± 0.4	55.0 ± 0.8
Acidic species	29.7 ± 0.4	30.1 ± 0.8	28.4 ± 0.5	33.0 ± 1.0

*Note*: Peak areas were determined using GXII reviewer and represent the mean of three technical replicates. Error bars represent ±1 *SD* of the mean.

**Table 4:** Purity analysis of intact mAb

Average of % relative amount	Feed strategy 4	Feed strategy 5	Feed strategy 6	Feed strategy 7
Peak 1	97.0 ± 0.2	96.3 ± 0.3	97.1 ± 0.3	96.9 ± 0.4
Peak 2	89 ± 3		91 ± 3	

*Note*: The average apparent size of the mAb peak is ~181 kDa. Peak areas were determined using GXII reviewer and represent the mean of three technical replicates. Error bars represent ±1 *SD* of the mean.

**Table 5:** Purity analysis of reduced mAb

	Feed strategy 4		Feed strategy 5		Feed strategy 6		Feed strategy 7	
	% Light	% Heavy	% Light	% Heavy	% Light	% Heavy	% Light	% Heavy
Peak 1	32.3 ± 0.6	66.2 ± 0.6	32.3 ± 0.6	65.9 ± 0.3	32.3 ± 0.5	66.5 ± 0.5	32.2 ± 0.5	66.4 ± 0.4
Peak 2	33 ± 1	37.1 ± 05	—	—	32.4 ± 0.7	46.4 ± 0.5	—	—

*Note*: The average apparent size of the light chain is ~29 kDa and the heavy chain is ~67 kDa. Peak areas were determined using GXII reviewer and represent the mean of three technical replicates on a mass basis. Error bars represent ±1 *SD* of the mean.

**Figure 4:**
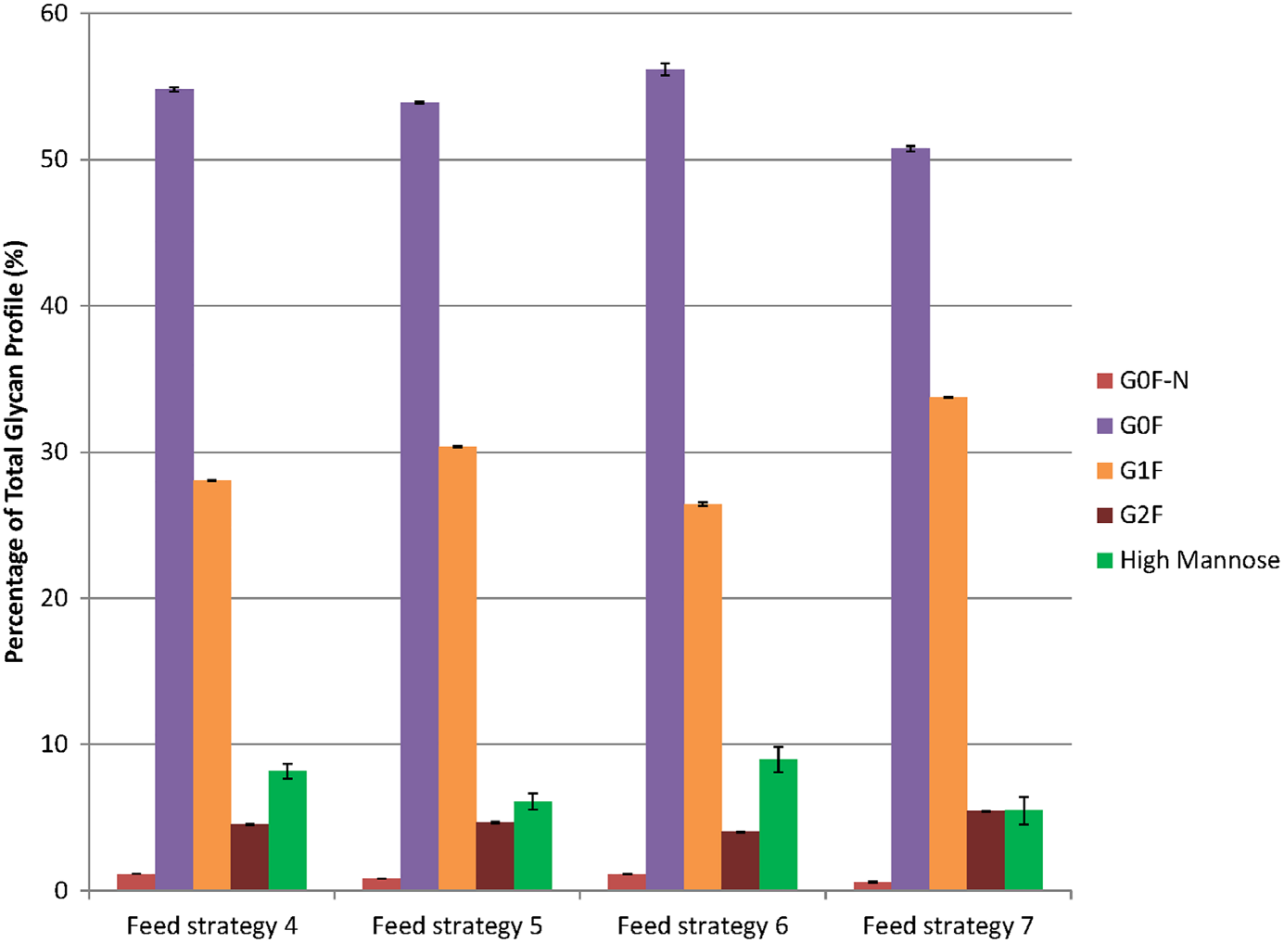
Final glycan profiles of mAb products. The final mAb products from the four bioreactor cultures supplemented with amino acids were characterized for the glycan profile. Feed strategies 4 and 6 both started with amino acid blend A, while feed strategies 5 and 7 used amino acid blend B

Likewise, the amino acid supplementation strategies had small, but statistically significant, effects on product quality from the standpoint of the glycan profile (Figure 4). The amino acid feeds that resulted in increased VCD and longer batch age performance (Feed strategies 4 and 6) also resulted in higher amounts of high mannose species production and lower amounts of terminal galactosylation (G1F and G2F). The main peaks and secondary peaks had highly similar glycan profiles, so only the main peaks are shown in Figure 4. Collectively, our protein structural analysis illustrates the importance of understanding how process parameters and bioreactor nutrients can affect product quality, as in this case where a favorable increase in VCD performance results in a potentially less favorable glycan profile outcome (with less galactosylation and increased high mannose glycoform amounts).

Due to the impact of amino acid blend supplementation on the glycan profiles of the produced antibodies, we used modeling approaches to better understand how amino acid consumption patterns are correlated to glycan outcomes. Univariate methods alone would not allow us to discover trends in the data that associate amino acid consumption patterns with glycan outcomes, so we employed multivariate approaches. We used batch data from all seven feeding strategies that we performed in this study to increase our statistical power for detecting significant relationships. Principal component analysis (PCA) was applied to allow us to aggregate a large amount of data across bioreactor runs and determine specifically which amino acids drive the observed changes.

**Figure 5:**
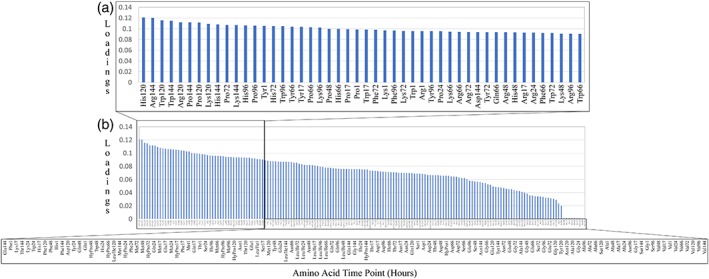
Multivariate data analysis of amino acid time point effect on glycan profile. The individual amino acid time points (hours) are shown in decreasing order of loading, which represents the correlation between the data point and final glycan outcomes. The highest loading indicates the highest correlation. (a) Subset of the most significant amino acid time points, those with loading values of ≥0.09. (b) The whole distribution of amino acid time points

The PCA included both the individual amino acid concentrations and their time‐dependent profiles to allow for all data to be considered and analyzed simultaneously, providing greater statistical power relative to a univariate comparison. The PCA model was developed including concentrations of 19 amino acids sampled at nine timepoints (1, 17, 24, 48, 66, 72, 96, 120, 144 hr) from the nine total bioreactor runs (five baseline runs, four experimental amino acid supplementation runs). Due to the differences in amino acid supplementation times, this approach allows us to understand how differences in the amino acid supplementation time might have affected the productivity of the bioreactor in terms of product quality. The first principal component captured 80% of the overall variability in the calibration dataset and 70% in the cross‐validation, which suggests that a relatively large amount of variability from the dataset can be captured by the first principal component. Because the aim of the PCA in this study is dimensionality reduction by identifying potentially critical amino acids during manufacturing, the loadings for the first principal component were further examined. The loading represents the impact of an amino acid at a certain time point on the overall variability of the dataset. Shown in Figure 5b, the amino acid time points have been presented in a decreasing order of association with glycan outcome (left to right). Each amino acid data time point features the hour of measurement, so each amino acid also has the same number of time points. Based on the Pareto principal, amino acid time points that have loadings greater than 0.09 are identified to be more important in the overall dataset, with this subset shown in Figure 5a. There are 48 sampling points that have loadings of more than 0.09, containing seven amino acids: Arg, His, Lys, Phe, Pro, Trp, and Tyr. In other words, among the 19 amino acids included in the PCA, the changes of these seven identified amino acids contribute to most of the glycan profile variability.

**Figure 6:**
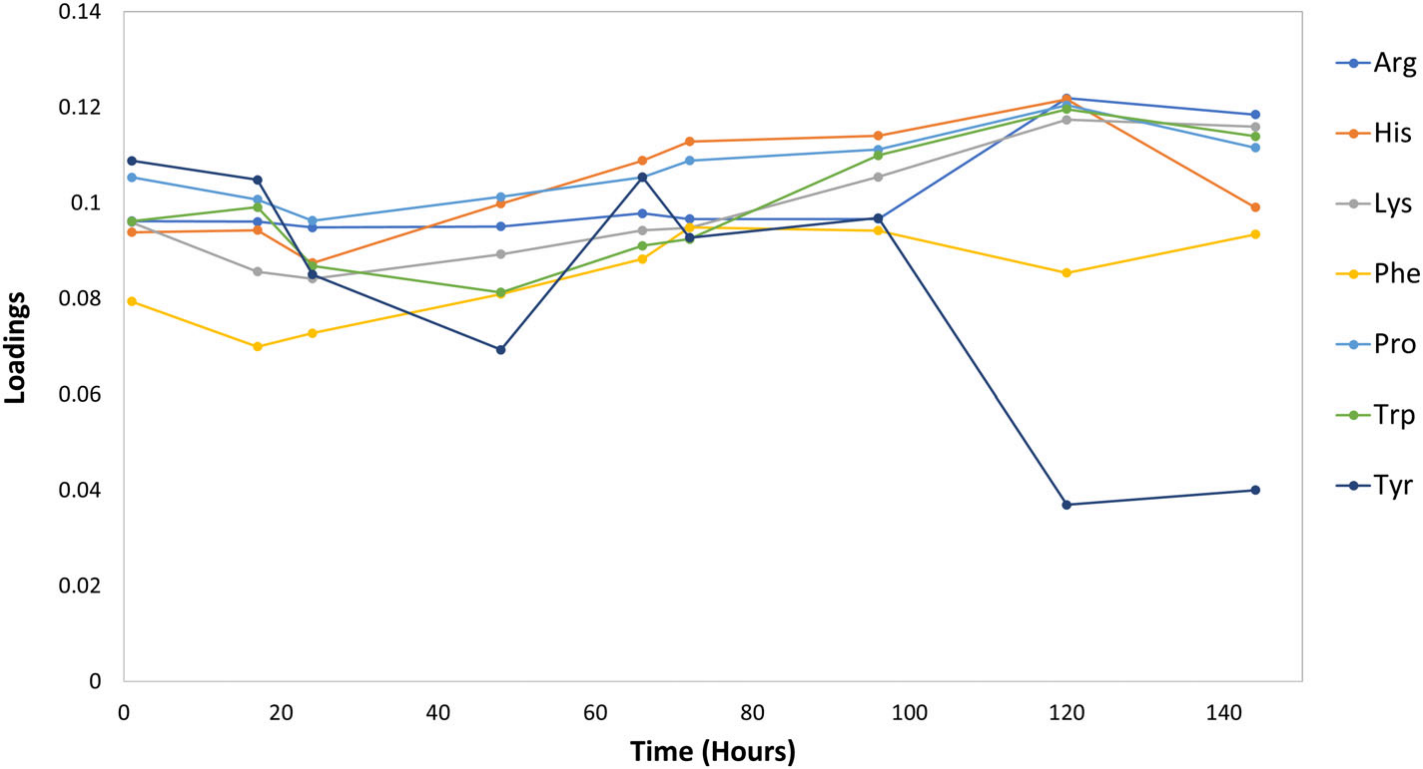
Time dependence of the amino acid correlation with final glycan profile outcomes. The most significant amino acids were mapped over batch age time (hours) to reveal time‐dependent trends for correlation between each constituent and glycan profile. While most of the amino acids increased with significance over time, tyrosine decreased over the final 48 hr when the first amino acid supplementation events occurred

Figure 6 demonstrates that the loadings of the identified seven amino acids vary over time and that the changes of the loadings are amino acid‐dependent. For example, there is a general decline in glycan profile association of all the seven amino acids during the first 24 hr of batch time. Following hour 24, a steady increase of loadings was observed for Arg, His, Lys, Phe, and Pro up to hour 140. Next, the timing of the amino acid supplementation strategy might have an effect on the increase of amino acid correlations with glycan profiles, as most of the amino acid supplementation events occurred between the 80‐hr and 120‐hr time points, during which the loadings of Arg, Pro, Trp, and Lys were found to steadily increase. Conversely, between hours 100 through 140 the impact of Tyr had a sudden drop and became not significant. The fluctuation observed for the tyrosine dependence for glycan outcome may be a consequence of its supplementation during that timeframe (as part of amino acid blend A). Tyrosine was supplemented at 127 hr and 119 hr in feed strategies 4 and 6, respectively, and the tyrosine correlation to glycan profile dropped during the 120 and 140 time points.

## 4 CONCLUSIONS

Amino acids are a critical component for biomanufacturing of protein products due to their role as the building blocks of the biotherapeutic, as well as other functions they can provide such as an alternative energy source. Despite this, the majority of amino acids are not monitored during a standard bioreactor run, with the exception being glutamine. To address this issue, as well as determine whether monitoring amino acids during upstream bioprocessing could be potentially important for either cellular growth or maintaining product quality, we experimented with amino acid supplementation strategies, developed a rapid at‐line amino acid quantification method, and used data modeling to predict which amino acids could have the largest effects on the final glycan profile.

We used five preliminary bioreactor runs to establish a baseline for amino acid consumption, both in batch mode and fed‐batch mode feeding strategies. Our rapid amino acid quantification method allowed us to analyze a large number of media samples from these runs and narrow down the amino acids of interest to eight: Tyr, Cys, Pro, Asn, Met, His, Trp, and Thr. We put these species together for multiple blend strategies and supplemented them into the bioreactors at critical points of the cell growth and nutrient consumption curve to observe their effects on the growth profile. We discovered that addition of Tyr, Cys, Pro, and Asn (blend A) resulted in a significant boost in VCD in the cultures, in one case even reversing a culture where the VCD was decreasing toward death based on historical trajectories. The baseline cultures that we performed before the experimental amino acid supplementation bioreactors indicate that once the VCD begins to decrease, the general health of the cultures has suffered (indicated by the viability) and cannot recover; despite this, the culture with feed strategy 6 (Figure 2e) was able to reach a new VCD maximum after amino acid blend A was administered. While it is unclear which of these four amino acids are responsible for the VCD effect, we are considering future studies to narrow down the amino acid responsible.

While amino acid supplementation may successfully prolong the runtime of a bioreactor, these strategies might have unknown effects on product quality. To address this, we analyzed the final mAb product from these bioreactor runs to determine their charge variant, size variant, and glycan profiles. We discovered that the amino acid blends did have small effects on the charge variant and glycan profiles. In the case of the glycan profile, we observed a small increase the percentage of high mannose glycoforms, which are generally undesirable. This is due to the faster clearance of these glycoforms as therapeutics, lowering their beneficial impact.^22^ The increase of these species is the result of incomplete processing of the glycan in the Golgi and is found when the culture is stressed, such as from high media osmolality or long culture times. Therefore, the increase in high mannose glycoforms could be due to the longer duration of the amino acid blend A supplemented cultures (240 hr vs. ≤216 hr for the non‐blend A cultures). Changes of this magnitude would not typically affect the drug molecule enough to cause it to fail specifications, although a cumulative set of changes that includes perturbed amino acid levels could additively cause the product to fail the acceptance criteria. Our findings indicate that prolonging the culture life using amino acids could impact product quality, something that would have to be addressed for maintaining performance standards.

In the final part of our study, we used PCA to see if any amino acids could be linked to the glycan outcome based on their consumption patterns by using data from all nine bioreactor runs. By comparing the cultures where amino acids were depleted and not replenished against those where they were, we can identify specific amino acid time points that are correlated with the glycan profiles. Using this approach in a time‐course‐oriented fashion, we established specific amino acids and time points which were most highly correlated. From all the amino acids analyzed, we narrowed down our pool of candidate species to Tyr, Pro, His, Trp, Arg, Lys, and Phe. Of these, four were among those that were actively supplemented in our blends (Tyr, Pro, His, and Trp). Our analysis revealed that the loadings for these amino acids changed over the course of the run, with most of them decreasing over the first 24 hr and increasing thereafter. Tyrosine was an interesting outlier from this trend, with a significantly lower loading over the final 40 hr of the culture life. We conclude that the timing of the amino acid supplementation between the 80‐hr and 120‐hr timepoints were a strong contributing factor to the trend lines for loadings correlations that we observed, such as the decrease discovered at the 120‐hr timepoint for tyrosine and the increases in loadings for Arg, His, Lys, Trp, and Pro over the same period.

The media within the bioreactor is a complex and continuously changing environment that has substantial effects on the cultured cells and their produced proteins. Due to the high number of nutrient variables that are present in the bioreactor that can all potentially affect product quality, future studies that utilize controlled and scheduled feeds (glucose/glutamine and amino acids) along with multivariate analysis will be necessary to more definitively establish the effect that amino acids have on the final protein. Multivariate analysis using data from all of the PAT instruments we have available (culture variables like temperature, pH, titer, and media variables such as glucose, glutamine, and lactate) along with our newly developed capabilities in measuring near real‐time amino acid concentrations will allow us to create a more comprehensive model and properly establish amino acid dependent effects on productivity and product quality.

With the increasing push toward process understanding and supporting real‐time analytics, better understanding of how concentrations of critical nutrients in the bioreactors impact productivity and product quality will become increasingly important. Better knowledge and informed supplementation strategies of the upstream process could provide increased yield of consistent quality and potentially avoid batch failures. This approach may involve near/real‐time amino acid monitoring and individualized supplementation strategies based on multivariate models similar to the procedure we have outlined in this research.

## NOTATION

ADCC: antibody‐dependent cellular cytotoxicity
CHO: Chinese hamster ovary
CQA: critical quality attribute
LC–MS: liquid chromatography mass spectrometry
mAb: monoclonal antibody
NIPALS: nonlinear iterative partial least squares
PAT: process analytical technology
PCA: principal component analysis
UV: ultraviolet
VCD: viable cell density

## DISCLAIMER

The article reflects the views of the authors and should not be construed to represent FDA views or policies.
